# Relationships Between Blood Pressure Reduction, Weight Loss, and Engagement in a Digital App–Based Hypertension Care Program: Observational Study

**DOI:** 10.2196/38215

**Published:** 2022-10-27

**Authors:** OraLee H Branch, Mohit Rikhy, Lisa A Auster-Gussman, Kimberly G Lockwood, Sarah A Graham

**Affiliations:** 1 Lark Technologies, Inc Mountain View, CA United States

**Keywords:** high blood pressure, obesity, weight loss, conversational artificial intelligence, lifestyle coaching

## Abstract

**Background:**

Home blood pressure (BP) monitoring is recommended for people with hypertension; however, meta-analyses have demonstrated that BP improvements are related to additional coaching support in combination with self-monitoring, with little or no effect of self-monitoring alone. High-contact coaching requires substantial resources and may be difficult to deliver via human coaching models.

**Objective:**

This observational study assessed changes in BP and body weight following participation in a fully digital program called Lark Hypertension Care with coaching powered by artificial intelligence (AI).

**Methods:**

Participants (N=864) had a baseline systolic BP (SBP) ≥120 mm Hg, provided their baseline body weight, and had reached at least their third month in the program. The primary outcome was the change in SBP at 3 and 6 months, with secondary outcomes of change in body weight and associations of changes in SBP and body weight with participant demographics, characteristics, and program engagement.

**Results:**

By month 3, there was a significant drop of –5.4 mm Hg (95% CI –6.5 to –4.3; *P*<.001) in mean SBP from baseline. BP did not change significantly (ie, the SBP drop maintained) from 3 to 6 months for participants who provided readings at both time points (*P*=.49). Half of the participants achieved a clinically meaningful drop of ≥5 mm Hg by month 3 (178/349, 51.0%) and month 6 (98/199, 49.2%). The magnitude of the drop depended on starting SBP. Participants classified as hypertension stage 2 had the largest mean drop in SBP of –12.4 mm Hg (SE 1.2 mm Hg) by month 3 and –13.0 mm Hg (SE 1.6 mm Hg) by month 6; participants classified as hypertension stage 1 lowered by –5.2 mm Hg (SE 0.8) mm Hg by month 3 and –7.3 mm Hg (SE 1.3 mm Hg) by month 6; participants classified as elevated lowered by –1.1 mm Hg (SE 0.7 mm Hg) by month 3 but did not drop by month 6. Starting SBP (β=.11; *P*<.001), percent weight change (β=–.36; *P*=.02), and initial BMI (β=–.56; *P*<.001) were significantly associated with the likelihood of lowering SBP ≥5 mm Hg by month 3. Percent weight change acted as a mediator of the relationship between program engagement and drop in SBP. The bootstrapped unstandardized indirect effect was –0.0024 (95% CI –0.0052 to 0; *P*=.002).

**Conclusions:**

A hypertension care program with coaching powered by AI was associated with a clinically meaningful reduction in SBP following 3 and 6 months of program participation. Percent weight change was significantly associated with the likelihood of achieving a ≥5 mm Hg drop in SBP. An AI-powered solution may offer a scalable approach to helping individuals with hypertension achieve clinically meaningful reductions in their BP and associated risk of cardiovascular disease and other serious adverse outcomes via healthy lifestyle changes such as weight loss.

## Introduction

The American Heart Association (AHA) defines high blood pressure (BP), called hypertension, as systolic BP (SBP) ≥130 mm Hg or diastolic BP (DBP) ≥80 mm Hg that remains elevated over time [1]. Nearly half of US adults have hypertension or are taking medication for hypertension, and only 1 in 4 have their BP under control [2]. Effective strategies to improve self-management of BP are critical since hypertension is a leading modifiable risk factor for cardiovascular disease [3], ranks as the leading cause of mortality in the United States [4], and is associated with a higher risk for other serious and costly conditions such as stroke, kidney disease, dementia, and eye damage [5]. In a large meta-analysis, lowering SBP by at least 5 mm Hg reduced the risk of major cardiovascular events by 10% even at normal (<120 mm Hg) and high-normal (120-129 mm Hg) values of SBP [6].

The AHA recommends home monitoring of BP as a part of self-management for all people with hypertension because it provides a better estimate of BP under “normal” conditions and may help improve BP control [7]. However, meta-analyses have provided strong evidence that BP improvements are related to cointerventions involving individually tailored coaching support in combination with self-monitoring, with little or no effect of self-monitoring alone [7,8]. Coaching can provide personalized support for increasing health-promoting lifestyle behaviors that are known to reduce BP, such as reaching and maintaining a healthy weight, eating a healthy diet, limiting alcohol consumption, avoiding smoking, adhering to prescribed medications, and being physically active [9]. Compared to a healthy weight, prior research has attributed an estimated 32% excess risk of hypertension to being overweight and 47% to being obese [10]. There is a well-characterized linear relationship between SBP and BMI, with a higher prevalence of hypertension in individuals in higher BMI classes [11]. Weight loss is a particularly important focus for individuals with hypertension who are overweight or obese because it is associated with improvements in BP control [12,13].

The US Preventive Task Force recommends moderate-to-high-intensity behavioral coaching for adults with cardiovascular risk factors including high BP and being overweight or obese [14]. However, behavioral coaching is most effective when it includes personalized content and feedback as well as frequent and timely interactions [15,16]. This type of coaching is highly time and resource intensive and may be difficult to achieve via human coaching models. Fully digital programs powered by artificial intelligence (AI) represent one solution for hypertension care that combines self-monitoring with highly personalized, automated coaching. An AI-powered coaching platform enables the delivery of continuous, synchronous coaching and feedback and offers a scalable, high-touch, and long-term solution to help people make lifestyle changes and sustain healthy behaviors. However, there is little evidence of the effectiveness of AI-powered solutions for facilitating reductions in BP and body weight for individuals with hypertension.

This observational study assessed changes in BP and body weight following participation in a fully digital hypertension care program powered by AI. This program used self-monitoring of BP coupled with conversational AI delivered on a participant’s smartphone to coach participants in lowering their BP, losing weight, and making other healthy lifestyle changes. The primary study objective was to evaluate the change in BP over time (baseline, 3 months, and 6 months), with secondary objectives of assessing changes in weight and associations of BP and weight change with AI-powered coaching and activities. The primary hypothesis was that participants with elevated or greater SBP (ie, ≥120 mm Hg) at baseline would lower their SBP on average by at least 5 mm Hg, which is commonly considered a clinically meaningful threshold for reduced cardiovascular disease risk [6,17]. We expected a greater reduction for participants with a higher starting SBP. The secondary hypothesis was that a greater reduction in BP and weight loss would be associated with greater participation in AI coaching and activities within the mobile app.

## Methods

### Participants and Recruitment

**Figure 1:**
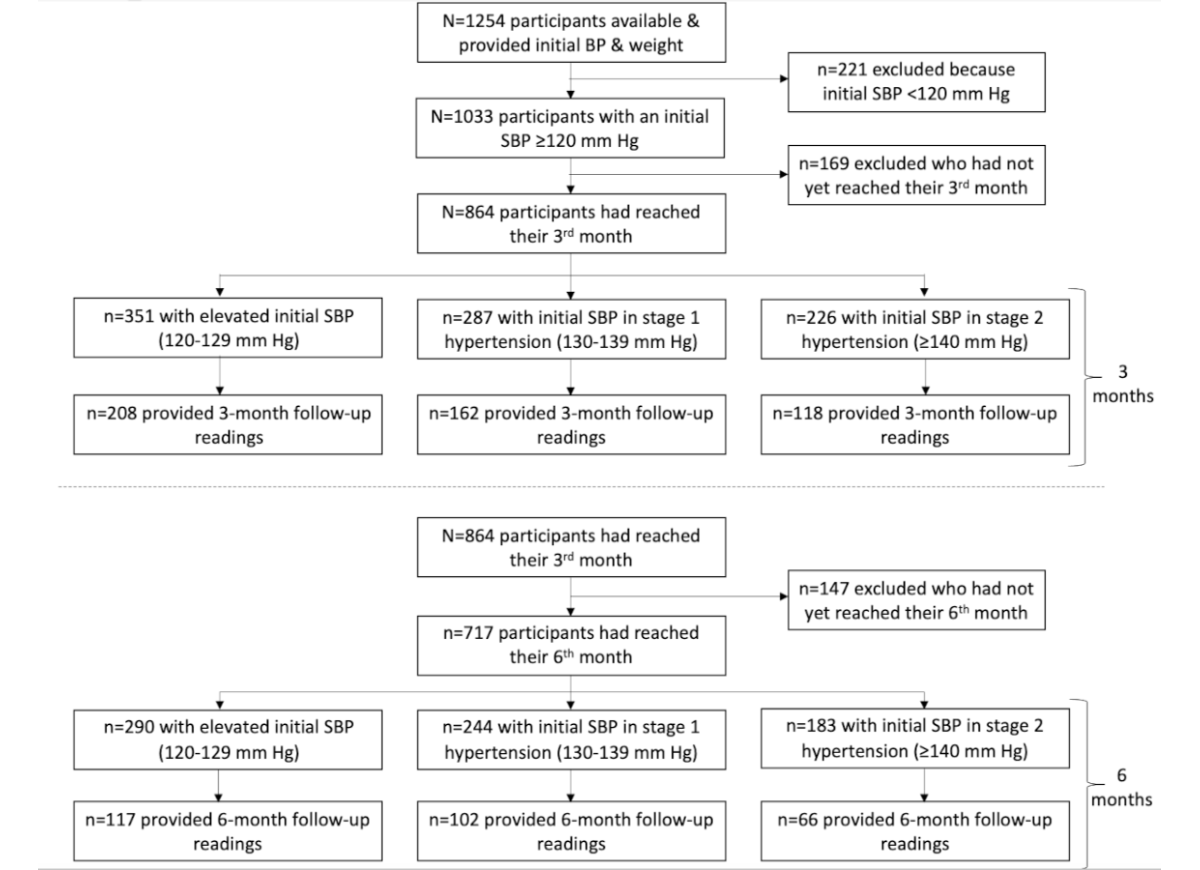
Flow of participants through the study. Participants categorized based on SBP as elevated, hypertension stage 1, or hypertension stage 2. Participants with readings at 3 and 6 months could be BP or weight. BP: blood pressure; SPB: systolic blood pressure.

This was a study of participants enrolled on a rolling basis (beginning January 1, 2019, and concluding on November 4, 2021) in a commercially available program called Lark Hypertension Care offered via existing partnerships between the company and health insurance providers, employers, and other organizations. The program recruits eligible participants via direct referrals from health plans or digital advertising through email campaigns and social media platforms such as Facebook. All participants received a link via SMS text messages that prompted them to download the mobile app, agree to the app’s privacy policy, and give permission to use their deidentified data for research. Included program participants were ≥18 years of age at enrollment, English speaking, owned an Android or iPhone smartphone, and had a respective health plan that identified them as having hypertension or being at risk for hypertension. Excluded participants did not provide initial BP or body weight readings; had an initial SBP reading <120 mm Hg, indicating that their BP was controlled; or had not yet reached at least their third month in the program (see Figure 1). A subset of participants had also reached their sixth month in the program for analysis of BP and weight change at 6 months.

### Ethical Considerations

The study received exemption status from Advarra Institutional Review Board (protocol #Pro00047181) for retrospective analysis of previously collected and deidentified data.

### Description of the Hypertension Care Program

The hypertension care program consisted of educational lessons and fully automated, personalized coaching on healthy lifestyle behaviors powered by conversational AI. Conversational AI technologies facilitate humanlike interactions between a robot (computer) and a human via a text-based interface. See Multimedia Appendix 1 [1,14,18-22] for a detailed description. After enrollment, participants completed a brief orientation on how to obtain accurate BP measurements, set medication reminders, and select an optional weight loss goal. Participants then progressed through weekly educational lessons spread over 26 weeks.

Participants in the program could opt to receive connected devices (digital BP cuff or weight scale) to measure BP and weight and could enter BP data in a variety of ways in the app, including wirelessly or manually. If using a connected BP monitor, participants could take a measurement through a guided coaching exchange and sync the measurement immediately. Those who already had a home BP cuff could use their existing device and manually enter BP readings in the app. Regardless of the measurement method, participants received detailed instructions on taking at-home BP measurements, as outlined by the AHA [18]. The program has built-in safety mechanisms: in the case of extremely high readings (SBP >180 mm Hg or DBP >110 mm Hg) or low readings (<90 mm Hg or <60 mm Hg) and symptoms like dizziness, the AI coach prompts participants to seek assistance or call their medical provider and assists them in taking these actions.

### Primary Outcome of Change in SBP

The primary outcome was the change in SBP from the start of the program to 3 and 6 months, respectively. Starting BP was a participant’s average measurement within the first week of the program, and 3- and 6-month BPs were a participant’s average measurement during the third and sixth month in the program, respectively. A few participants had readings that occurred soon after the sixth month, and we included these data points as well to maximize the sample size available for analysis. This was necessary for a real-world study where participants were not aware that they were supposed to provide readings at a particular time point. We considered a clinically meaningful improvement to be a drop of ≥5 mm Hg at any time point. We also conducted subgroup analyses on participants classified as having elevated SBP at baseline (SBP 120-129 mm Hg), stage 1 hypertension (SBP 130-139 mm Hg), and stage 2 hypertension (SBP ≥140 mm Hg). We assessed corresponding changes in DBP based on starting SBP classification.

### Secondary Outcomes of Change in Body Weight and Associations With Program Engagement

The secondary outcome was the percent weight change at month 3 and month 6, respectively. We calculated the percent weight change at 3 months as follows: (first weight – 3-month nadir weight)/first weight. We calculated the percent weight change at 6 months as follows: (first weight – 6-month nadir weight)/first weight. We removed any abnormal weigh-ins indicating a weight loss or gain rate of >7 lbs/week unless confirmed by the user to be a correct measurement. We assessed associations between the change in SBP and body weight at month 3 with participant demographics, characteristics, and engagement metrics using 2 separate regression models. For the regression with change in SBP as the dependent variable, independent variables included participant demographics (age, sex), characteristics (initial BMI, starting SBP, percent weight change), and program engagement metrics (number of sessions with the AI coach, number of BP measurements). For the regression with percent weight change as the dependent variable, independent variables included participant demographics (age, sex), characteristics (initial BMI, starting SBP), and program engagement metrics (number of sessions with the AI coach, number of weight measurements). We examined these associations at month 3 instead of month 6 due to the larger sample size available at month 3 (for statistical power) and since most of the change in BP occurred by month 3 and was maintained through month 6 for participants who provided both measurements.

### Statistical Analyses

We conducted all statistical analyses in RStudio 4.0.5. We compared participant demographic and characteristic data between subgroups categorized by baseline SBP classification. We used paired *t* tests to evaluate the change in BP and body weight between each pair of time points (baseline, 3 months, 6 months) to maximize the sample size available for each comparison (more participants had made it to 3 months in the program [N=864] compared to 6 months [n=717]). We conducted two separate regression analyses: (1) a multiple logistic regression to assess the effects of participant demographics, characteristics, and program engagement on participants’ likelihood of achieving a clinically meaningful drop in SBP of ≥5 mm Hg by month 3 in the program; and (2) a linear regression to assess the effects of participant demographics, characteristics, and program engagement on percent weight change by month 3 in the program. We conducted these analyses separately to consider each outcome independently and because not all participants had both BP and weight data available at 3 months. Engagement variables in both regressions had no issues of multicollinearity; all variance inflation factors in both models were <2. The results of the regression analyses suggested that percent weight change was a mediator of the relationship between program engagement (number of sessions with the AI coach) and SBP drop, so we conducted an exploratory full mediation analysis [23] to confirm this observation. The a priori α was ≤.05 for all statistical tests.

## Results

### Participant Demographics and Characteristics

There were significant differences across participants based on the SBP category for initial BMI (Table 1). Initial BMI increased, on average, by 1 unit for each increase in the SBP classification category.

**Table 1:** Baseline demographics and characteristics for all participants and grouped by starting systolic blood pressure.

	BP^a^ category					*F* test/ chi-square (*df*)^b^		*P* value
	All, mean (SE)^c^	Elevated, mean (SE)^c^	Stage 1 hypertension, mean (SE)^c^	Stage 2 hypertension, mean (SE)^c^				
Age (years)	51.5 (0.34)	52.3 (0.54)	51.2 (0.55)	50.7 (0.70)	1.9 (2,847)		.14	
Initial BMI (kg/m^2^)	34.0 (0.25)	33.2 (0.37)	34.2 (0.39)	35.2 (0.56)	5.4 (2,861)		.005	
No. of BP readings included in baseline BP	3.6 (0.11)	3.5 (0.16)	3.9 (0.21)	3.3 (0.19)	2.6 (2,861)		.07	
Baseline systolic BP (mm Hg)	134.5 (0.38)	125.0 (0.15)	134.3 (0.17)	149.5 (0.60)	1506.0 (2,861)		<.001	
Baseline diastolic BP (mm Hg)	85.3 (0.30)	81.4 (0.38)	85.4 (0.43)	91.4 (0.63)	110.5 (2,861)		<.001	
Female sex^c^	500/861 (58.1)	216/351 (61.5)	162/286 (56.6)	122/224 (54.5)	3.2 (2)		.21	
White race^c^	370/499 (74.1)	157/207 (75.8)	128/168 (76.2)	85/124 (68.5)	2.7 (2)		.26	
Taking BP meds^c^	530/578 (91.7)	228/245 (93.1)	156/177 (88.1)	146/156 (93.6)	4.3 (2)		.12	

^a^BP: blood pressure.

^b^Chi-square is applicable only to female sex, White race, and taking BP meds. For the other demographics, *F* test is applicable.

^c^Mean (SE) here is not applicable to categories of female sex, White race, and taking BP meds; for these categories, data are expressed as n/N (%).

### Changes in BP

Participants provided a mean of 3.6 (SE 0.1) BP readings for the calculation of average starting BP, a mean of 15.0 (SE 1.2) readings for the calculation of average BP at month 3, and a mean of 17.0 (SE 1.6) BP readings for the calculation of average BP at month 6.

There was a significant overall drop of –5.4 mm Hg in mean SBP following 3 months (*t*=9.5_348_; *P*<.001; 95% CI –6.5 to –4.3), and no change in SBP from 3 to 6 months for those who provided readings at both time points (*t*=0.7_139_; *P*=.49; Table 2). Participants with a starting SBP classified as hypertension stage 2 had the greatest change in SBP at both time points, with a drop of –12.4 mm Hg (SE 1.2 mm Hg) by month 3 and a drop of –13.0 mm Hg (SE 1.6 mm Hg) by month 6.

Approximately half of the overall sample achieved a clinically meaningful SBP drop of ≥5 mm Hg by month 3 (178/349, 51%) and by month 6 (98/199, 49.2%). The drop in SBP resulted in 47.6% (166/349) of participants lowering their SBP by at least 1 classification category (eg, hypertension stage 2 to hypertension stage 1; hypertension stage 1 to elevated) by month 3.

**Table 2:** Change in blood pressure from baseline to 3 and 6 months.

		Change in BP^a^ at 3 months, Δmean (95% CI)^b^	*t* test (*df*)	*P* value	With ≥5 mm Hg SBP^c^ drop at month 3, n/N (%)	Change in SBP at 6+ months, Δmean (95% CI)^b^	*t* test (*df*)	*P* value	With ≥5 mm Hg SBP drop at month 6, n/N (%)
**SBP^b^ (mm Hg)**									
	Overall	–5.4 (–6.5 to –4.3)	9.5 (348)	<.001	178/349 (51.0)	–5.3 (–6.9 to –3.6)	6.3 (198)	<.001	98/199 (49.2)
	120-129	–1.1 (–2.5 to 0.4)	1.5 (148)	.14	51/149 (34.2)	0.6 (–1.7 to 2.8)	–0.5 (85)	.62	25/86 (29.1)
	130-139	–5.2 (–6.8 to –3.7)	6.6 (106)	<.001	53/107 (49.5)	–7.3 (–9.8 to –4.8)	5.8 (64)	<.001	36/65 (55.4)
	≥140	–12.4 (–14.9 to –10.0)	10.2 (92)	<.001	74/93 (79.6)	–13.0 (–16.2 to –9.8)	8.2 (47)	<.001	37/48 (77.1)
**DBP^d^ (mm Hg)^e^**									
	Overall	–1.3 (–2.1 to –0.5)	3.1 (348)	.002	N/A^e^	–1.2 (–2.3 to –0.2)	2.3 (198)	.02	N/A^f^
	Elevated	0.6 (–0.6 to 1.7)	–1.0 (148)	.34	N/A	1.0 (–0.6 to 2.5)	–1.2 (85)	.22	N/A
	Stage 1	–0.9 (–2.4 to 0.5)	1.3 (106)	.21	N/A	–2.4 (–4.2 to –0.7)	2.8 (64)	.007	N/A
	Stage 2	–4.6 (–6.2 to –3.0)	5.8 (92)	<.001	N/A	–3.5 (–5.7 to –1.3)	3.2 (47)	.002	N/A

^a^BP: blood pressure.

^b^Negative Δ values indicate a drop in BP and positive values an increase.

^c^SBP: systolic blood pressure.

^d^DBP: diastolic blood pressure.

^e^DBP categories based on initial SBP classification: elevated, hypertension stage 1, or hypertension stage 2.

^f^N/A: not applicable.

### Associations With BP Drop and Weight Change

Results of the multiple logistic regression for BP revealed associations of participant demographics, characteristics, and program engagement metrics with the likelihood of achieving a clinically meaningful drop of ≥5 mm Hg in SBP by month 3. The overall regression was statistically significant (log-likelihood –152.3; McFadden's pseudo *R^2^*=0.18; *P*<.001. Starting SBP, initial BMI, and percent weight change at month 3 were significantly associated with the likelihood of achieving a drop of ≥5 mm Hg in SBP (Table 3).

**Figure 2:**
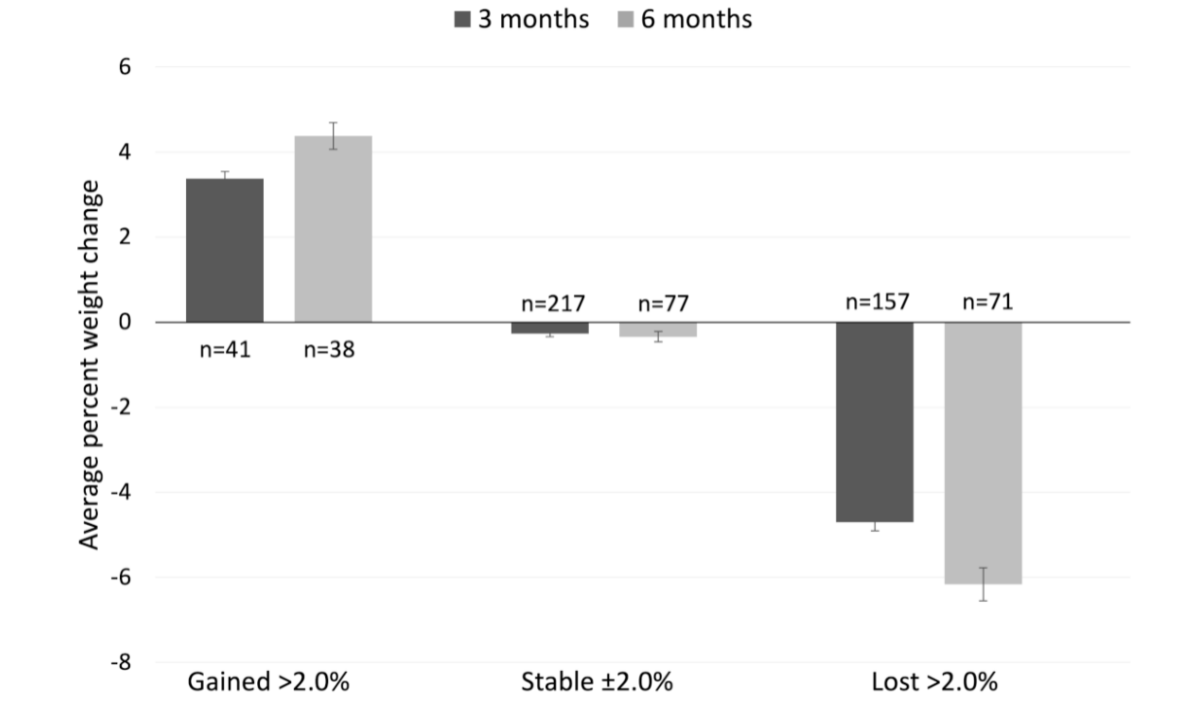
The number of participants and mean (SE) for each category of weight change: gained, stable, and lost for the 415 participants who provided weigh-ins at 3 months and the 186 who provided weigh-ins at 6 months.

Of the participants who provided weigh-ins in the third month, 90.1% (374/415) remained weight stable or lost weight over the first 3 months of the program (Figure 2).

Results of the multiple linear regression for weight change revealed associations of participant demographics, characteristics, and program engagement metrics with the magnitude of percent weight change by month 3. The overall regression was statistically significant (*F*_7,397_=5.97; *R^2^*=0.10 *P*<.001). Initial BMI, the number of sessions with the AI coach, and the number of weigh-ins recorded in the first 3 months were significantly associated with percent weight change (Table 4).

The 2 regression models together demonstrated that percent weight change at month 3 was significantly associated with the likelihood of achieving a ≥5 mm Hg drop in SBP, and program engagement variables were significantly associated with the magnitude of percent weight change but not SBP drop. Thus, it was important to consider whether percent weight change acted as a statistical mediator between program engagement and SBP drop. The results of the mediation analysis indeed demonstrated that the effect of program engagement on the drop in SBP was fully mediated by percent weight change at month 3.

**Figure 3:**
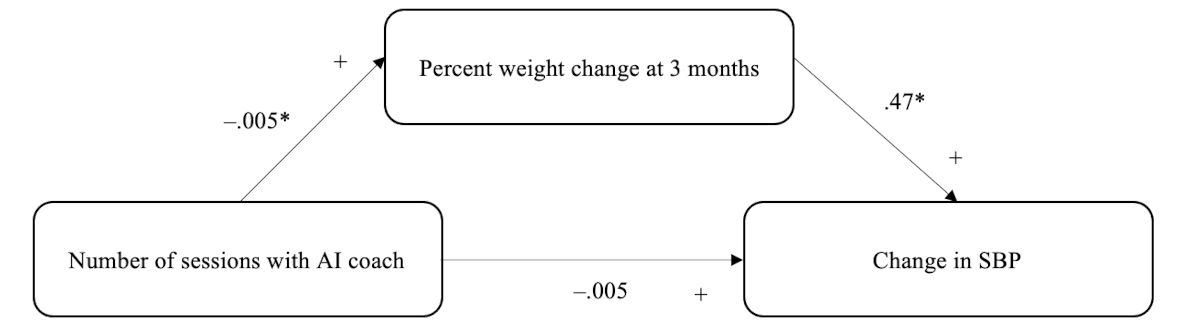
Percent weight change at 3 months as a mediator of the relationship between program engagement and change in SBP. A negative coefficient indicates that a greater number of sessions with the AI coach was significantly related to greater weight loss (represented by a negative sign). The positive coefficient for the significant relationship between percent weight change and change in SPB is because drops (improvements) in both are represented with a negative sign. AI: artificial intelligence; SBP: systolic blood pressure. *Unstandardized coefficients and significance level ≤.001.

As Figure 3 illustrates, the regression coefficient between percent weight change at month 3 and the drop in SBP was significant, even though the regression coefficient between the number of sessions with the AI coach and the drop in SBP was not. Although the total effect was therefore not significant, this is not considered a requirement for statistical mediation [23]. We tested the significance of the unstandardized indirect effect of the number of sessions with the AI coach on the change in SBP that occurred via the mediator percent weight change at month 3 using 1000 bootstrapped samples. The bootstrapped unstandardized indirect or average causal mediation effect was –0.0024 (95% CI –0.0052 to 0; *P*=.002).

**Table 3:** Regression results of the likelihood of lowering ≥5 mm Hg in SBP by 3 months (n=268)^a^.

Variable	Standardized coefficient (β)	SE	*Z* value	*P* value
Constant	.12	0.14	0.87	.38
Age	–.03	0.15	–0.24	.81
Male sex	–.19	0.14	–1.31	.19
Initial BMI (kg/m^2^)	–.56	0.15	–3.57	<.001
Percent weight change by 3 months^b^	–.36	0.15	2.43	.02
Starting SBP^c^	1.11	0.18	6.13	<.001
No. of sessions with AI^d^ coach in first 3 months	–.04	0.16	–0.27	.79
No. of BP measurements recorded in first 3 months	.05	0.16	0.28	.78

^a^Included participants had to have both blood pressure and weight data available at 3 months.

^b^A negative sign for weight change indicates greater weight loss.

^c^SBP: systolic blood pressure.

^d^AI: artificial intelligence.

**Table 4:** Regression results for associations of participant demographics, characteristics, and program engagement with the dependent variable percent weight change. For the dependent variable, weight change, a negative value indicates weight loss (n=400)^a^.

Variable	Standardized coefficient (β)	SE	*t* value	*P* value
Constant	–1.57	0.15	10.26	<.001
Age	.05	0.17	–0.30	.77
Male sex	.09	0.15	–0.59	.56
Initial BMI (kg/m^2^)	–.48	0.16	3.02	.003
Starting SBP^b^	.17	0.15	–1.09	.27
No. of sessions with AI^c^ coach in first 3 months	–.62	0.16	3.81	<.001
No. of weight measurements recorded in first 3 months	–.44	0.16	2.78	.006

^a^Larger sample size due to removed requirement for blood pressure data at 3 months.

^b^SBP: systolic blood pressure.

^c^AI: artificial intelligence.

## Discussion

### Principal Findings

The primary aim of this study was to assess changes in SBP following participation in an AI-powered hypertension care program. We further assessed changes in weight and associations of BP and weight change with participant characteristics and program engagement. In support of the primary hypothesis, participation in the AI-powered hypertension care program was associated with clinically meaningful reductions in SBP over 3 to 6 months, with larger drops observed in the subgroups of participants classified as stage 1 or 2 hypertension at baseline. More than half of all participants achieved a clinically meaningful drop in SBP of ≥5 mm Hg. The percentage of participants that achieved a clinically meaningful reduction was higher for those classified as hypertensive at baseline, with 79.6% (74/93) of participants with a starting SBP ≥140 mm Hg achieving a drop of ≥5 mm Hg by month 3 in the program. Percent weight change at month 3 was significantly associated with program engagement and the likelihood of achieving a clinically meaningful drop in SBP, and percent weight change mediated the relationship between program engagement and change in SBP.

The overall average reduction in SBP of –5.4 mm Hg in this study corresponded to roughly half of the participants lowering their initial SBP classification by at least 1 classification category by month 3. Key aspects of the program included reminders to monitor BP, medication adherence support, personalized in-the-moment feedback about progress, hypertension-specific nutrition coaching, stress-reduction coaching, and educational material about hypertension. Prior research has shown that this type of personalized and multifaceted intervention is critical for success in digital hypertension care programs [24]. Compared to individuals with normotensive BP, individuals with treated but uncontrolled hypertension are at a higher risk of cardiovascular, cerebrovascular, and all-cause mortality [25]. Lowering BP is of well-established importance for those with hypertension stage 2; however, lowering BP for individuals with elevated BP or hypertension stage 1 is also clinically meaningful because absolute reductions in the risk of stroke, major cardiovascular events, and cardiovascular and all-cause mortalities have been shown to be progressively lower with a lower attained value of SBP [26].

The observed change in systolic BP in this study is comparable to the drops reported in published human coach–led behavioral lifestyle interventions. In the meta-analysis by Tucker et al [8], only 1 of 5 interventions that investigated self-monitoring alone showed a significant drop in SBP, with a pooled average of −1.0 mm Hg (95% CI −3.3 to 1.2). In contrast, self-monitoring with intensive coaching support via counseling or telecounseling showed statistically significant drops in SBP compared to a control group, with a pooled average reduction of –6.1 mm Hg (95% CI −9.0 to −3.2). The present study provides new evidence that members of a fully digital program powered by AI coaching experienced improvements in their BP while enrolled in the program.

Participants in this study were obese on average, with the overall initial BMI of 34.0 kg/m^2^ falling into class I obesity [27]. There was a 1-unit increase in initial BMI for each increasing classification of starting SBP, with participants classified as elevated having an average BMI of 33.2 kg/m^2^, hypertension stage 1 an average BMI of 34.2 kg/m^2^, and hypertension stage 2 an average BMI of 35.2 kg/m^2^ (class II obesity). Given that weight loss is associated with improvements in BP control [12,13], weight loss was a particularly important target for participants in this study. Percent weight change at month 3 was significantly associated with the likelihood of achieving a clinically meaningful drop in SBP of ≥5 mm Hg. Participants with a higher initial BMI were less likely to achieve this clinically meaningful drop. However, in the regression with percent weight change as the dependent variable, participants with a higher initial BMI lost a greater amount of weight. Taken together, it appears that there were some participants with a higher initial BMI that did not achieve a clinically meaningful reduction in BP. However, for the larger number of users analyzed in the weight change regression, having a higher initial BMI was associated with a greater percent weight loss.

There are multiple reasons for the observed relationship between percent weight change and achieving a clinically meaningful drop in SBP in this study. There are well-established physiological benefits of weight loss for hypertension, such as improvement in insulin sensitivity and a decrease in sympathetic nervous system activity and inflammation [12,13,28]. However, given that percent weight change was significantly associated with program engagement and statistically mediated the relationship between program engagement and SBP drop in this study, it may also be that percent weight change was an indicator of those participants who were more closely adhering to the recommendations of the AI coach and adopting the healthy lifestyle changes and behaviors (eg, diet, exercise) that are also known to lower BP [9,29]. Indeed, prior research has linked weight loss to behaviors such as frequent tracking of exercise and weight [30].

### Study Strengths and Limitations

This was a single-arm study, preventing any determination of cause and effect. However, participants were real-world users of a commercially available digital health program designed for hypertension management; thus, this study provides evidence for the effectiveness of an AI-powered behavioral coaching program for lowering BP in the target population of interest. Participants were not required to provide socioeconomic information (eg, income, education), which limited insight into potential socioeconomic disparities. Although retention was lower than that in clinical efficacy studies, this is expected for digital health [31], and retention was substantially higher than what is commonly reported for similar programs in the literature [32]. Prior investigations have demonstrated that self-monitoring along with self-titration of medications in collaboration with a treating physician yields robust drops in BP and good retention [33]. Engaging physicians in the member experience could be one way to improve member retention. The exploratory mediation analysis had some inherent limitations: without the ability to infer causal relationships or directionality from the results of this study, this was not “true” mediation. Given the timing of the measurements, we cannot state that engagement caused weight loss, which then caused BP reductions. However, the alternative model (engagement → BP reduction → weight loss) was not significant, which supports the directionality of the relationship between engagement, percent weight loss, and BP change proposed in this study.

### Future Directions

This study was an important first step in demonstrating changes in BP and body weight that occurred during a fully digital hypertension management program. Although we had information on other important BP management strategies at baseline (eg, medication status), we did not have the ability to track changes made to participants’ care management that might have occurred during the duration of the study. In future investigations, we intend to examine interactions between program participation and additional aspects of care. Evaluating medication adherence is a new feature within the app, and future investigations will examine whether participating in a fully digital hypertension management program results in improved adherence to prescribed medications. Finally, we did not separately consider coaching sessions per topic area (eg, diet) in the regression analyses due to collinearity issues. However, certain types of coaching may be more important than others. We plan to explore the different factors related to AI coaching in future investigations.

### Conclusions

Members enrolled in a fully digital hypertension care program with coaching powered by AI who provided BP readings during their third and sixth months of program participation achieved clinically meaningful reductions in SBP. The magnitude of the drop depended on starting SBP, with participants classified as hypertension stage 2 experiencing the greatest drop. Most participants remained weight stable or lost weight by month 3 in the program, and the percent weight change at month 3 was significantly associated with program engagement and the likelihood of achieving a drop of ≥5 mm Hg in SBP. Taken together, these results provide formative evidence that members enrolled in an AI-powered hypertension care program who remain engaged experience clinically meaningful reductions in their systolic BP and body weight.

## Appendix

Multimedia Appendix 1 Description of the Lark Hypertension Care Program.

## Abbreviations

AHA: American Heart Association
AI: artificial intelligence
BP: blood pressure
DBP: diastolic blood pressure
SBP: systolic blood pressure
