# Supplementary material for: Relationships Between Blood Pressure Reduction, Weight Loss, and Engagement in a Digital App–Based Hypertension Care Program: Observational Study
Source: JMIR Form Res. 2022 Oct 27;6(10):e38215. doi: 10.2196/38215 (PMC9650575; doi:10.2196/38215)
Supplement: Multimedia Appendix 1 [file formative_v6i10e38215_app1.docx]

### **Multimedia Appendix 1: Description of the Lark Hypertension Care Program**

***Program Overview***

Lark Hypertension Care is a mobile app-based coaching program that provides personalized counseling and education focused on self-management of high blood pressure (BP). Members receive education on why measuring BP is important, what a healthy BP range is, and more detailed coaching to help each member identify trends and connections between their behaviors and BP readings. The application is compatible with both Android and iOS devices. The program follows the American Heart Association (AHA) guidelines for the management of high BP [1]. Educational content consistent with these guidelines is developed by Lark’s Health Committee and lifestyle coaches and tailored to unique member needs. The Health Committee includes the following Lark employees: a medical doctor, a nutritionist expert, five clinical research PhDs, two psychologists, a rehabilitation exercise specialist, and an expert in machine learning data science. The Health Committee also includes external advisors who challenge and review the program content, direction, and member feedback and experiences.

Members have 24/7 access to educational content and personalized coaching support provided in-app to encourage healthy lifestyle choices and assist users in working toward the adoption of healthy behaviors. The Hypertension Care program educational lessons focus on topics endorsed by the AHA including nutrition, physical activity, weight loss, sleep, and stress reduction (ex: Figure S1). Lark is unique among digital preventive care programs because the program is powered by conversational Artificial Intelligence (AI). Lark AI coaching models the coaching behavior of a human coach and provides educational curriculum, feedback, insights, and summary information on user progress. Using proprietary AI algorithms, Lark coaching provides information, feedback, and support in real-time when it is most relevant and helpful to the member. Lark coaching follows best practices in behavior change science to motivate members to build the knowledge and skills they need to take small steps that lead to significant and lasting behavior change over time [14].


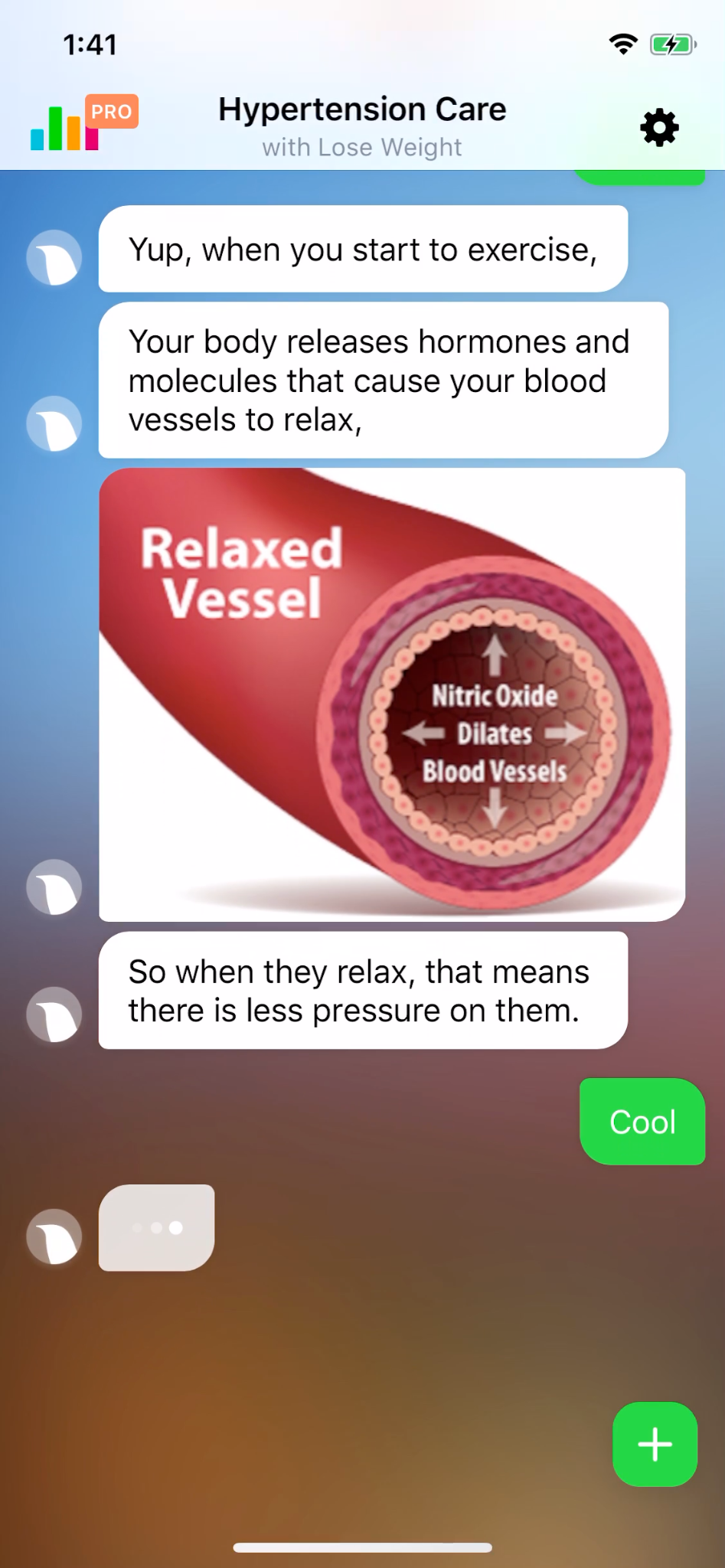


Figure S1. Example educational lesson content.

***Program Fees/Incentives***

Lark members do not pay for the Hypertension Care program directly; it is a covered benefit under their insurance plan. Potential members are informed about the program through outreach from their insurance provider and/or Lark. Digital advertising (e.g., Facebook) helps facilitate awareness of this covered service. Eligible members download the program to their Android or iOS smartphone and have 24/7 access to the Lark AI coach and other application features. Members are eligible for the program if they are ≥18 years of age at enrollment, English-speaking, own an Android or iPhone smartphone, and their respective health plan identifies them as having hypertension or being at risk for hypertension. After enrollment, members complete a brief orientation on how to obtain accurate BP measurements, set medication reminders, and select an optional weight loss goal. Incentives are not a standard practice in the Lark programs, though some members may receive small monetary rewards for completing surveys or generating data that helps to further refine the Lark experience.

***Educational Lessons***

The Hypertension care program is designed for members to interface with educational content delivered in digestible amounts. Educational content is organized around 26 week-long lessons called “missions” with weekly topics shown in Table S1. Members are encouraged to check in with lesson content once per day.

Table S1: Lark Hypertension Care Weekly Lesson Topics

| **Lesson #** | **Lark Title/Topic** |
| --- | --- |
| 1 | Activity for Low Blood Pressure |
| 2 | Eat Well |
| 3 | Sodium and Hypertension |
| 4 | Burn Calories |
| 5 | Get More Active |
| 6 | Track Your Food |
| 7 | Track Your Activity |
| 8 | Shop and Cook |
| 9 | Manage Stress |
| 10 | Find Time for Fitness |
| 11 | Cope with Triggers |
| 12 | Heart Health |
| 13 | Take Charge of your Thoughts |
| 14 | Support Network |
| 15 | Eat Well Away From Home |
| 16 | Maintaining Motivation |
| 17 | Taking Ups and Downs In Stride |
| 18 | Sedentary Time |
| 19 | Stay Active |
| 20 | Stay Active Away From Home |
| 21 | Lifestyle For a Healthier You |
| 22 | More About Carbs |
| 23 | Enjoy Healthy Food |
| 24 | Get Enough Sleep |
| 25 | Eyes on the Prize |
| 26 | Wellness For Life |

***Secondary Program Goals***

BP reduction is the primary goal for Hypertension Care members. Thus, the primary program goal is set to Manage Hypertension at the start of the program. Members may also elect optional secondary goals at the start of the program: Stress Less or Weight Loss. Additionally, once the member has completed the 26-lesson “Manage Hypertension” education portion of the program (Table S1 above), they may switch their primary focus to Stress Less, Lose Weight, or Tobacco Cessation (described further under Conversational AI Coaching below).

***Conversational AI Coaching***

The Lark AI coach interacts with members and engages them in text-based conversations delivered within the mobile application. Though Lark is designed to mimic a human coach with its conversational AI and compassionate tone, users are informed that their smartphone-based coach is digital. The AI coach offers synchronous communication, meaning that members can engage in conversations at any time upon opening the app. Members also receive daily “nudges” or reminders to encourage them to engage with the platform. The AI coach provides members with positive reinforcement after logging healthy behaviors, gives immediate personalized feedback on logged data, and presents daily and weekly summaries of program progress (e.g., Figure S2). Members can also view their data and progress at any point in the program through a personalized data dashboard. Content provided by the Lark AI coach is tailored to unique member needs with programming logic that considers factors including where the member is in the program, information the member has recently input (e.g., logging a meal, activity, weight, sleep), and member goals. If a member indicated they are interested in losing weight or reducing stress, they receive weekly check-ins and monthly check-ins about progress toward their weight goal and/or for stress reduction.


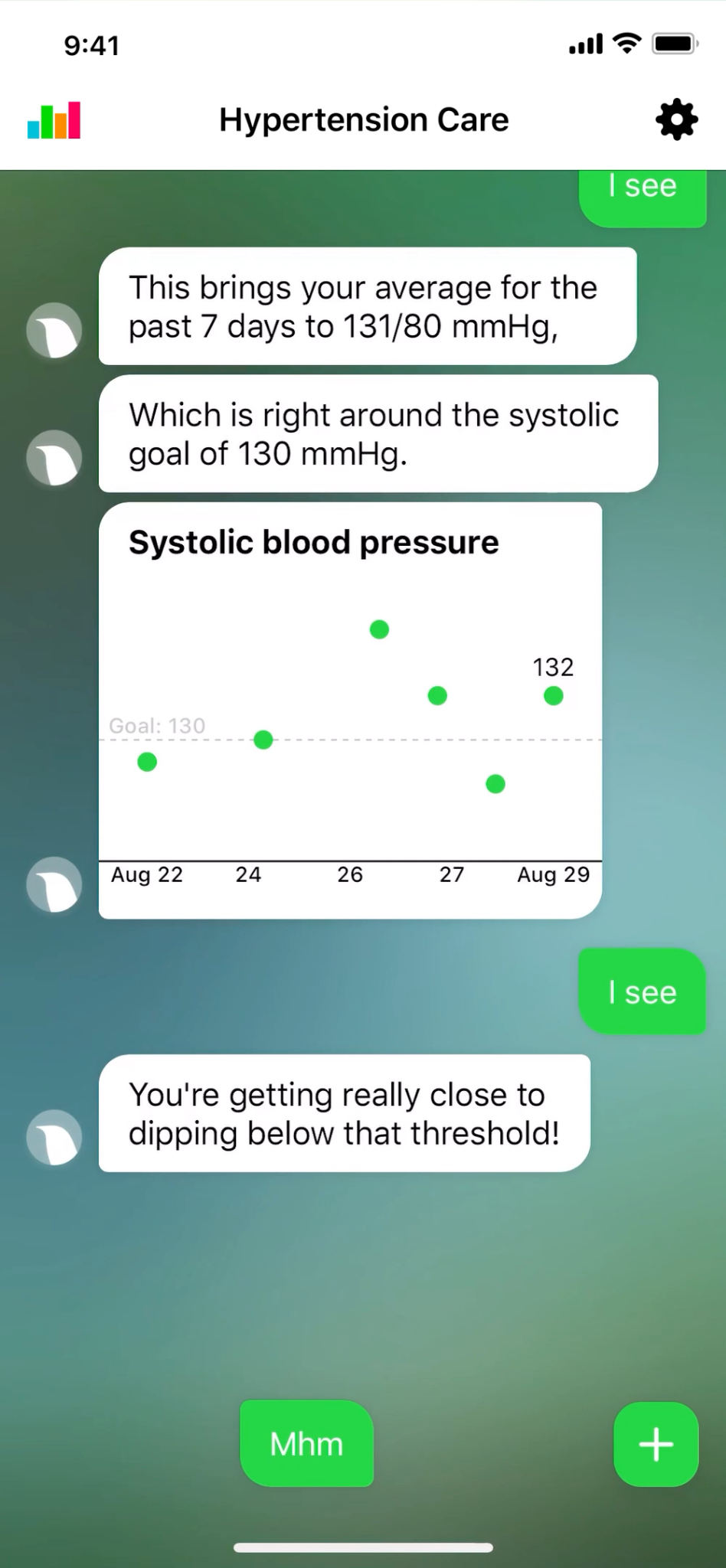


Figure S2. Example of blood pressure tracking within the mobile application.

Members receive specific instructions on how to measure their BP accurately at home. These guidelines follow the information provided by the AHA on home BP measurement [18]. Coaching specific to BP first involves one week of intensive engagement collecting daily readings to quickly assess the member’s hypertensive state and variability in readings. After the first week, and a minimum number of BP readings, the member receives an initial assessment of their BP baseline and where they fall in relation to the thresholds for hypertension set forth by the AHA. Thereafter, the Lark coach provides daily measurement reminders and periodic BP check-ins. If measurements indicate a new trend or outlier, the coach engages the member to determine preceding conditions or triggers like a high sodium meal or less sleep the previous night. The BP goal for the program is to maintain daily readings under 130/80 mm Hg. The program has built-in safety mechanisms; in the case of extremely high readings (SBP >180 mmHg or DBP >110 mmHg) or very low readings (<90 mmHg or <60 mmHg) and symptoms like dizziness, the AI coach prompts members to seek assistance or call their medical provider and assists them in taking these actions.

***Connected Devices***

Members can opt to receive connected devices (digital BP cuff and/or weight scale) to measure BP and weight and can enter BP data in a variety of ways in the app, including wirelessly or manually. If using a connected BP monitor, members can take a measurement through a guided coaching exchange and sync the measurement immediately. Those who already have an approved home BP cuff can use their existing device and manually enter BP readings in the app. Regardless of the measurement method, members receive detailed instructions on taking at-home BP measurements, as outlined by the AHA [18].

***Medication Adherence***

The Lark AI coach asks members if they are taking medication, assesses baseline medication adherence, and helps members set up reminders. Members may toggle the setting “using medication” on or off under customized coaching settings. If it is toggled “on” then members can set up to four medication reminders. The Lark coach periodically checks in with members to update their medication plan. If barriers to medication adherence are identified, the member is encouraged to call and inform their healthcare provider. Members are prompted to rate their medication adherence and reasons for it at three-month intervals during the program.

***Blood Pressure Specific Nutrition Coaching***

Rather than focusing solely on calories, Lark follows current research and recommendations from the Health Committee and focuses on the quality of foods eaten. Nutrition coaching emphasizes choosing a nutritious diet like the Mediterranean or DASH diet and consuming foods that are associated with lower BP. Examples of foods that the Lark AI coach recommends include vegetables, whole grains, dietary fiber, fruit, lean proteins, and healthy fats. Members are encouraged to minimize their consumption of foods or nutrients associated with high BP including saturated and trans fats, sugar-sweetened foods and beverages, total starches, and fried foods. Lark’s meal logging system is powered by natural language processing and enables users to quickly type in foods they consumed with an estimated amount (e.g., half a turkey sandwich). When users enter foods and beverages, Lark provides real-time, personalized feedback that includes education on food components, suggestions for improvements, and/or insights based on a user’s personal data (Figure S3). Users earn green badges each time they achieve the daily goal for a dietary component (food or nutrient) that Lark tracks.


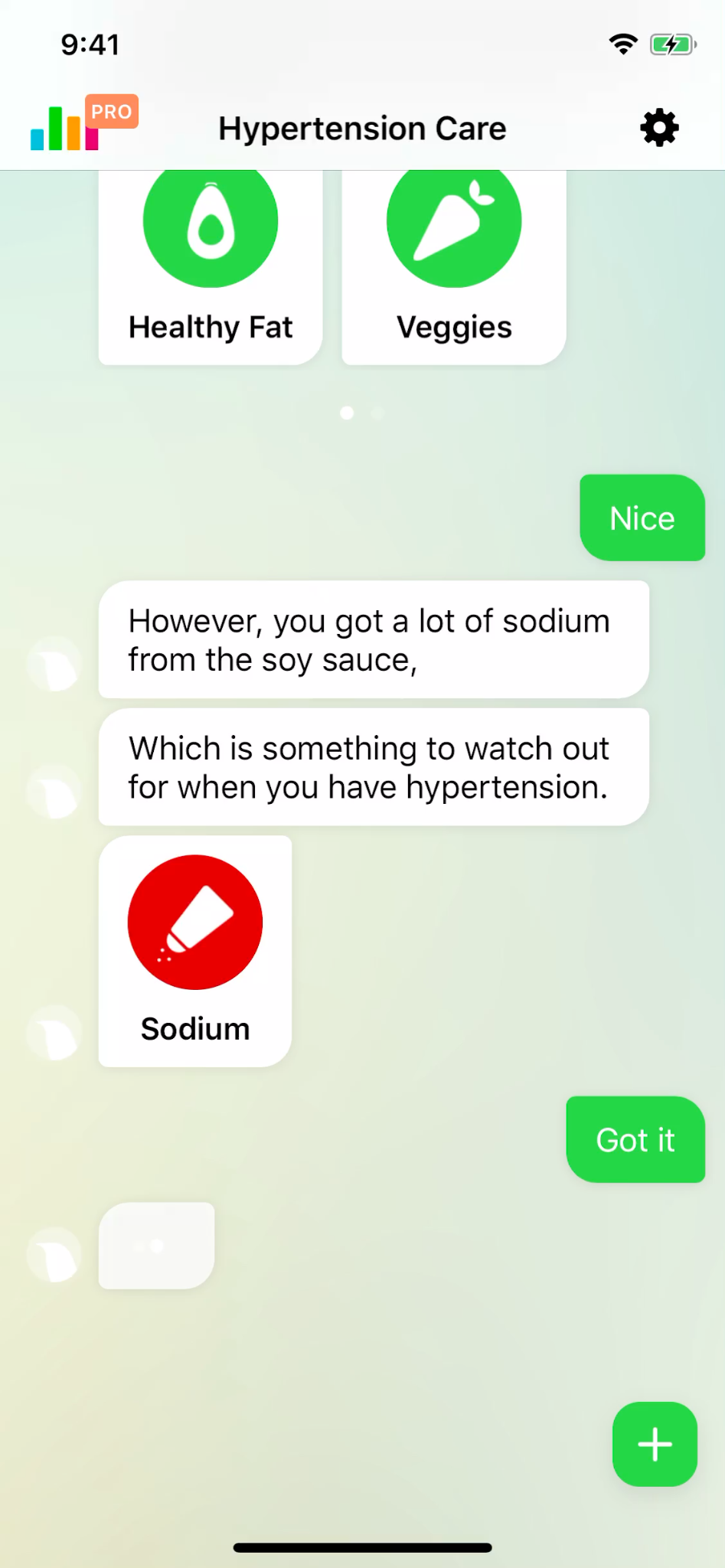


Figure S3. Example of in-the-moment coaching on blood-pressure-specific nutrition.

***Blood Pressure Specific Physical Activity Coaching***

Physical activity coaching emphasizes how physical activity helps lower BP and the best types of activity for individuals with hypertension. Members learn about the benefits of moderate-intensity cardiovascular exercise and are assisted in planning for engaging in at least 150 minutes of moderate or brisk physical activity each week and minimizing sedentary time. Members can manually log exercise sessions within the Lark app, and Lark also detects activity from the phone’s motion sensors or 3rd party apps like iOS Health kit and Google Fit. Lark provides daily feedback on activity totals and alerts members to days when activity is below or above average.

***Coaching Incorporates Evidence-Based Theories of Behavioral Change***

Lark uses theories of behavior change including cognitive behavioral therapy [19], positive psychology [20], and motivational interviewing [21] to help members make lifestyle changes that become habits and reduce health risks. Table S2 shows examples of coaching principles and strategies used by the Lark AI coach during coaching exchanges with members.

Table S2: Examples of Principles and Strategies Consistent with Theories of Behavior Change Used in the Lark Program

| **Principle/Strategy** | **Example(s) in Lark Hypertension Care** |
| --- | --- |
| Goal setting | Set and monitor progress toward BP reduction and/or weight loss and daily physical activity goals |
| Problem solving | Identify barriers to healthy behaviors and develop positive action plans to overcome them |
| Immediate feedback | In-the-moment feedback when members log a meal or physical activity and summaries of daily and weekly progress |
| Self-monitoring | Nudges to encourage members to log meals and physical activity and weigh-in |
| Building confidence | Celebration of accomplishments (e.g., BP measurements in normal range) |
| Promoting motivation | Find social support; make new action plans (plan ahead) |
| Practicing positive thinking | Talk back to self-defeating thoughts with positive thoughts |
| Reducing stress | Suggest deep breathing exercises when facing a stressful situation |
| Making commitments | Ask members to commit to a goal (e.g., BP reduction, weight loss, smoking cessation) |
| Taking small steps toward goals | Ask members when they can incorporate physical activity into their day and which activity they may choose |

***Data Monitoring***

Lark monitors members’ data through a variety of sources including smartphone motion sensors, connected blood pressure cuff, connected weight scale, integrations with Apple Health and Google Fit, Lark’s proprietary Digital Nutrition Therapy food logger, and member text conversations and surveys. Information is analyzed in real-time, providing highly personalized, timely coaching and insights to members that help drive behavior change. These data are also available for analysis, as Lark members agree to a privacy policy releasing their de-identified data for research purposes.

***Safety and Escalations***

Harms associated with lifestyle interventions for BP reduction are rare and, when reported, not significantly different between intervention and control groups [14]. Lark does not replace regular visits with a healthcare provider. However, Lark does screen for medical conditions that need to be escalated to a member’s healthcare provider. The app also has additional screeners. For example, members take the PHQ2 depression screening survey [22], and if their score indicates that they are at risk (i.e., ≥3) they are directed to resources within their healthcare provider. Lark also performs quarterly screening via survey questions to determine if a member has become pregnant or sustained a severe illness, injury, or surgery that renders them unable to continue in the program.
